# Thickness-dependent thermoelectric power factor of polymer-functionalized semiconducting carbon nanotube thin films

**DOI:** 10.1080/14686996.2018.1500851

**Published:** 2018-08-13

**Authors:** Yoshiyuki Nonoguchi, Ami Takata, Chigusa Goto, Takuya Kitano, Tsuyoshi Kawai

**Affiliations:** a Division of Materials Science, Nara Institute of Science and Technology, Ikoma, Japan; b PRESTO, JST, Kawaguchi, Japan; c NAIST-CEMES International Collaborative Laboratory, CEMES-CNRS, Toulouse, France

**Keywords:** Thermoelectric transport, carbon nanotubes, conjugated polymers, morphology, spectroscopy, plasmon resonance, doping, 50 Energy Materials, 104 Carbon and related materials, 105 Low-Dimension (1D/2D) materials, 210 Thermoelectronics / Thermal transport / insulators, 505 Optical / Molecular spectroscopy

## Abstract

The effects of polymer structures on the thermoelectric properties of polymer-wrapped semiconducting carbon nanotubes have yet to be clarified for elucidating intrinsic transport properties. We systematically investigate thickness dependence of thermoelectric transport in thin films containing networks of conjugated polymer-wrapped semiconducting carbon nanotubes. Well-controlled doping experiments suggest that the doping homogeneity and then in-plane electrical conductivity significantly depend on film thickness and polymer species. This understanding leads to achieving thermoelectric power factors as high as 412 μW m^−1^ K^−2^ in thin carbon nanotube films. This work presents a standard platform for investigating the thermoelectric properties of nanotubes.

## GRAPHICAL ABSTRACT

**Figure:**
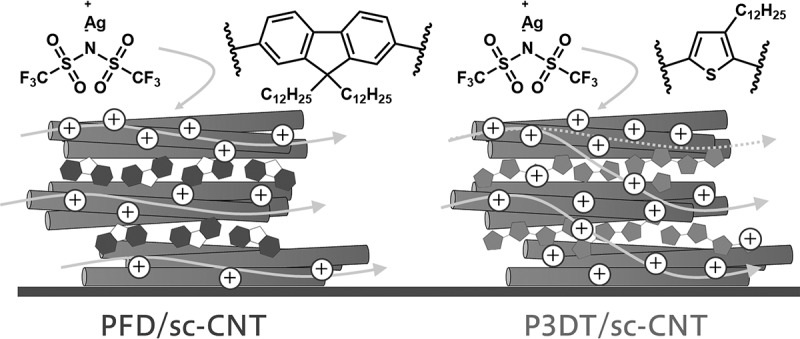

## 1. Introduction

Investigating thermoelectric transport in single-walled carbon nanotubes (SWNTs) deepens our understanding of their electronic properties as a quasi-one dimensional material and potential as energy harvesting materials [1]. The investigation of SWNTs transport requires us to address two practical issues. Firstly, most SWNTs are obtained as the ensembles of various structural forms, and their electronic types vary from metallic (m-) to semiconducting (s-), depending on chirality [2]. Many applications require separation of SWNTs with a certain electronic type, and considerable progress has been made recently in this area [3–7]. Secondly, the controlled doping that modulates the Fermi level energy, carrier density, and carrier polarity is similarly required. The Seebeck coefficient can be approximately expressed as a function of the gradient of density of state (DOS), *δDOS*/*δE* at the Fermi level. In this context, several researchers have recently achieved such a thermoelectric property modulation using field effect and electrochemical transistor techniques [8–10]. We have also demonstrated the amphoteric (both p-type and n-type) chemical doping of SWNTs, achieving ambient stability and precise doping level control [11–16].

Presently, challenges remain, including the construction of basic structure-property relationship – it is expected that thermoelectric properties vary depending on both primary structures (e.g. metallicity (chirality), defect density, atomic doping) and morphology (e.g. individuals/junctions, dopant distribution) [17–20]. In this context, it is important to experimentally link structural factors to thermoelectric properties. Among various methods, the separation of high-performance s-SWNTs was achieved using conjugated polymers as an extractant [5]. This technique has been further developed so that fluorene copolymers with various main and side chains, enable the selective separation of specific-diameter s-SWNTs [21]. The technical background above has recently been applied in the investigation of thermoelectric transport. Ferguson and Blackburn have very recently investigated thermoelectric transport in doped semiconducting SWNT thin films [1,22]. Their reported power factors were approximately five times larger than those of s-SWNT films prepared by other separation methods. These polymer-functionalized s-SWNT materials can therefore be recognized as the preferred platform enabling the investigation of structural effects on thermoelectric properties.

Herein, we seek to understand the factors limiting the transport properties of polymer-functionalized s-SWNT films. In this study, we examine the influence of film thickness and polymer species on the thermoelectric properties of the s-SWNT films. Such molecular factors are important for controlling not only structure separation mentioned above but also dopant miscibility. Poly(9,9-di-n-dodecylfluorenyl-2,7-diyl) (**PF12**) and poly(3-dodecylthiophene-2,5-diyl) (**P3DT**) were used for the separation of s-SWNTs with a diameter of approximately 1.0 nm. Thin films composed of polymer-wrapped s-SWNTs were fabricated by the filtration of stock dispersion and its transfer onto PET films. We then observe the thickness-dependent power factor when **PF12** is used for separation. We compare this result to the transport properties of **P3DT**-functionalized s-SWNT films, where almost no thickness dependence is observed. The effects of polymer dispersants on the thermoelectric properties are elucidated.

## 2. Experimental section

### 2.1. Materials

SWNTs (Raymor Industries Inc., RN020), **PF12** (Sigma-Aldrich Japan, Tokyo, Japan), **P3DT** (Sigma-Aldrich Japan, Tokyo, Japan), silver bis(trifluoromethanesulfonyl)imide (AgTFSI, Sigma-Aldrich Japan, Tokyo, Japan), sodium deoxycholate (**SDOC**, >96% purity, Wako Pure  Ltd., Osaka, Japan), deuterium oxide (D_2_O, 99.9%, Wako Pure Chemical Industries Ltd., Osaka, Japan) and toluene (Spectrochemical Analysis Grade, Wako Pure Chemical Industries Ltd., Osaka, Japan) were used as received.

### 2.2. Extraction of s-SWNTs

Typically, the dispersion of s-SWNTs was prepared with the ratio 8 mg SWNT/2.0–3.5 mg **PF12** (or 10 mg **P3DT**)/25 mL toluene. This mixture was then homogenized for 30 min using horn sonication (Q-125, QSONICA, Newtown, CT, USA) with a 1/4 in. tip operated at a duty cycle of 50% (each 5 s) at 12 W input power. The dispersion was cooled in an ice bath during sonication so that SWNTs were not overheated or severely damaged (Figure S1). This was then promptly followed by centrifugation at 10,000 rpm for 60 min (model 5500, Kubota, Tokyo, Japan). Aqueous **SDOC** dispersions, used for comparison with polymers, consisted of 8 mg SWNT and 25 mL 1% **SDOC**. Its dispersion was performed in a similar manner above.

### 2.3. Thin film fabrication

**Figure 1.**
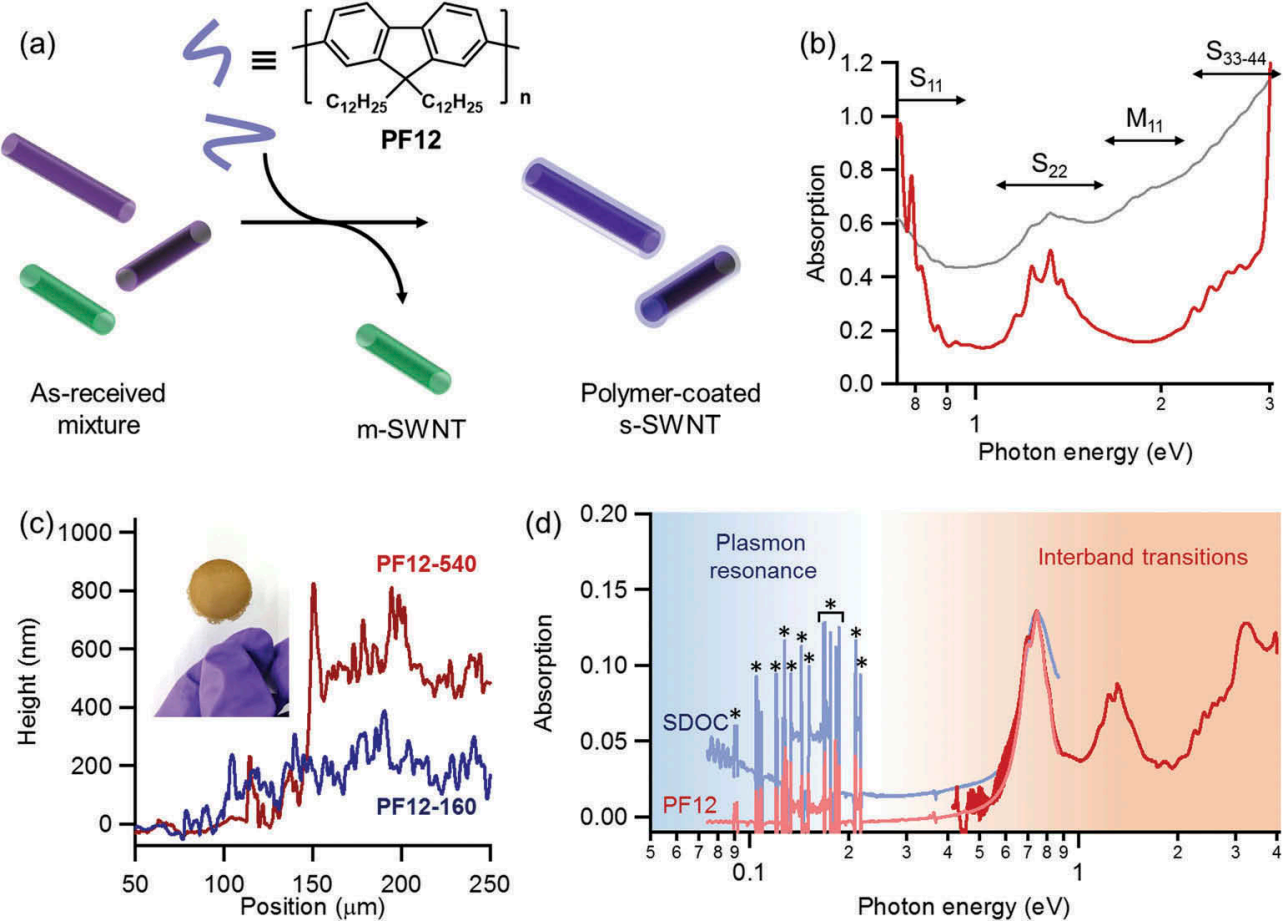
(a) Schematic of the separation of s-SWNTs from as-received mixture by conjugated polymers. (b) Absorption spectra of s-SWNTs dispersed by PF12 in toluene (brown line) and by **SDOC** in D_2_O (grey line). (c) Height profiles of transferred films on PET substrates. The inset photograph shows the reddish brown **PF12/SWNT-320** film on PET. (d) Absorption spectra of an s-SWNT film in the UV-Vis-NIR (brown) and NIR-MIR (pale red) regions. A pale blue line shows the MIR spectra of a SDOC-dispersed film as a reference. Sharp background spikes (*) in the MIR region (0.09–0.22 eV) are derived from the vibration modes of PET.

The dispersion was filtered onto membrane filters with 0.2 μm pores, followed by vacuum drying at 80 °C for 30 min. Thin films made wet with 2–3 drops of toluene were then transferred from the membranes onto polyethylene terephthalate (PET) substrates by rubbing (the inset of Figure 1(c)).

### 2.4. Chemical doping

For chemical p-doping, s-SWNT films (200–600 nm in thickness, 17 mm in diameter) on PET substrates were immersed in 10 mL butanol solution containing AgTFSI at fixed concentrations (0–8 mg mL^−1^) for 5 min. After drying at a reduced pressure (GLD-051, ULVAC, Chigasaki, Japan), samples were transferred to measurement systems.

### 2.5. Characterization

Absorbance measurements were taken using a UV−vis−near-infrared (NIR) spectrophotometer (UV-3600plus, Shimadzu, Kyoto, Japan), and a Fourier transform infrared (FTIR) microscope (HYPERION 2000, Bruker Japan, Yokohama, Japan). Height profiles were measured using a step profiler (ET200, Kosaka Laboratory, Tokyo, Japan). Thermoelectric properties were evaluated using the ZEM-3M10 system (ADVANCE RIKO, Yokohama, Japan). Experimental errors were estimated considering the systematic error of measurement methods (e.g. *σ*, 10%; *α*, 15%).

## 3. Results and discussion

### 3.1. Extraction of s-SWNTs and their structure discrimination

S-SWNTs were extracted using **PF12** dissolved in toluene (Figure 1(a)). Polyfluorene derivatives are known to adsorb preferentially onto s-SWNTs via π-π interaction, which is widely used for the selective recognition and separation of s-SWNTs [5]. Furthermore, dodecyl (C_12_) side chains were chosen to dissolve s-SWNTs with relatively large diameters of approximately 1.0 nm. In the UV-Vis-NIR spectroscopy, three peak groups were observed (Figure 1(b)), attributed to the first (S_11_, > 1400 nm), second (S_22_, 800–1200 nm), and third (S_33_, < 600 nm) interband transitions between the van Hove singularity points [23]. Importantly, compared to **SDOC** D_2_O reference dispersion, the interband absorption of m-SWNTs (M_11_, 600–800 nm) was found to effectively decrease to its background level after **PF12** separation. Background absorption in Figure 1 is too strong to allow a 1% accuracy in evaluating the metallic component. The M_11_ band is relatively weak even in non-separated samples. The **PF12/s-SWNT** films showed the characteristic absorption band of **PF12** (~ 400 nm, ~ 3 eV, extinction coefficient ~ 10^5^ cm^−1^) [24]. Considering the extinction coefficient of polymers and SWNTs (~ 5 × 10^4^ cm^−1^), we then estimated the volume composition of **PF12** in the **PF12/s-SWNT** films as 23 ± 5%.

In order to evaluate transport properties, thin films composed of s-SWNTs were fabricated (the inset of Figure 1(c)). Their thickness was determined using a nanometre-resolution surface profiler, and sample films were denoted, for example, as **PF12/s-SWNT-160** for a 160 nm-thick s-SWNT film prepared using **PF12**. In the following section, we use s-SWNTs separated not only by **PF12** but also by **P3DT** (e.g. **P3DT/s-SWNT-180** depicted for a 180 nm-thick film in Figure 4.

**Figure 2.**
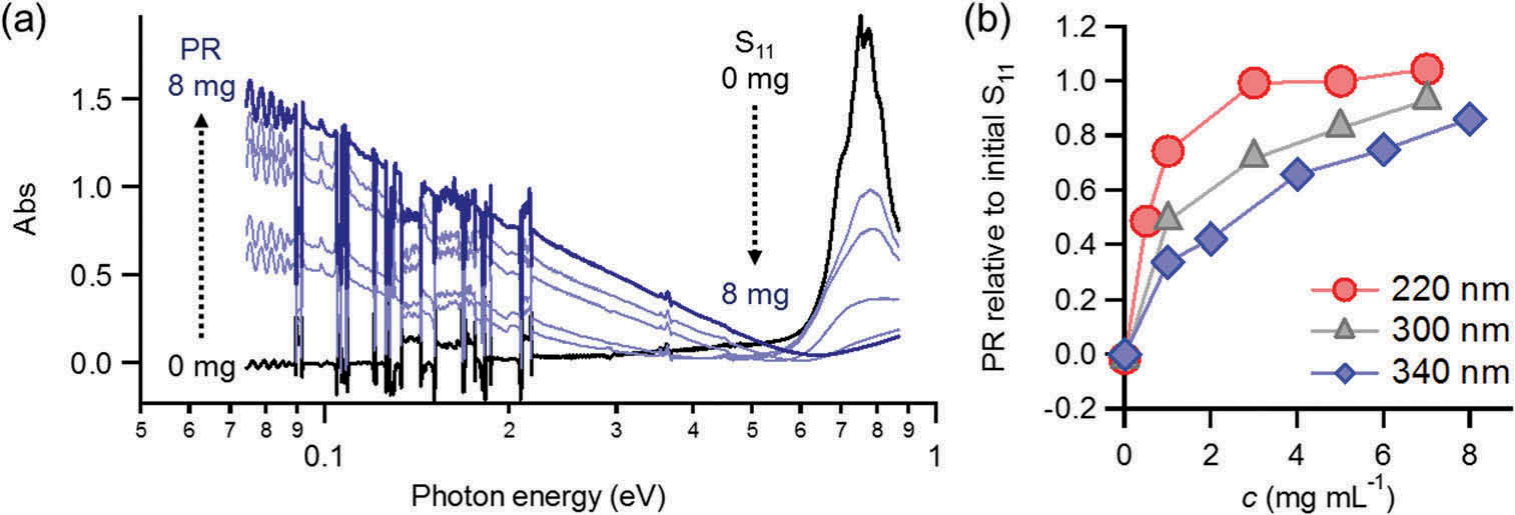
(a) Absorption spectra of **PF12/s-SWNT-340** before and after doping with AgTFSI up to 8 mg mL^−1^ in butanol. (b) Evolution of plasmon resonance (PR) band as a function of AgTFSI concentration for 220 nm-, 300 nm-, and 340 nm-thick **PF12/s-SWNT** films. The PR intensity at 0.1 eV is normalized by the initial S_11_ peak at 0.8 eV.

**Figure 3.**
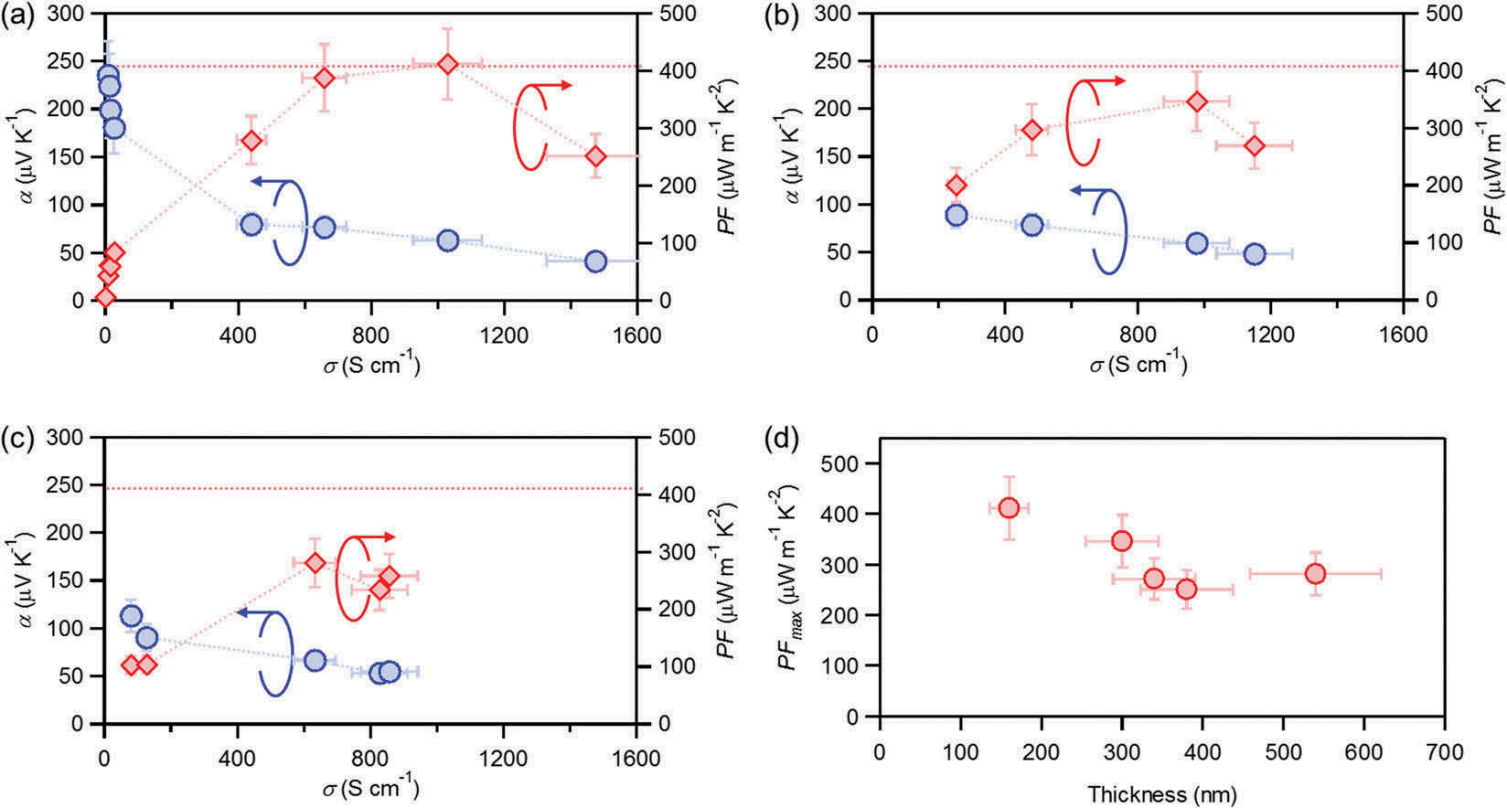
Thermoelectric properties of (a) **PF12/s-SWNT-160**, (b) **PF12/s-SWNT-300**, and (c) **PF12/s-SWNT-540** after AgTFSI doping with different concentration. (d) Optimized power factors as a function of film thickness.

**Figure 4.**
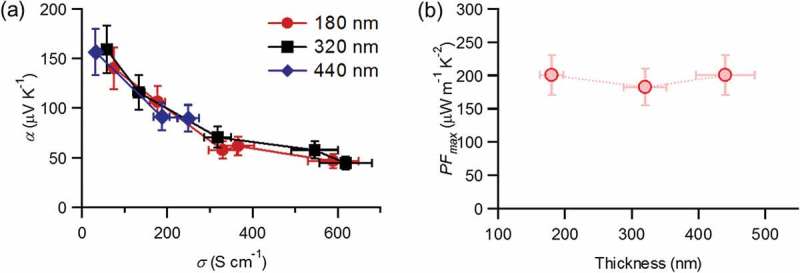
(a) Thermoelectric properties of **P3DT/s-SWNT-180, P3DT/s-SWNT-320**, and **P3DT/s-SWNT-440**. (b) Optimized power factors as a function of film thickness.

The fabricated films appeared semitransparent (the inset of Figure 1(c)), which enables optical absorption spectroscopy in a wide energy range (ultraviolet (UV) to mid-infrared (MIR)). This broadband spectroscopy could quantify the doping level. The structures of interband transitions were mostly preserved from those of s-SWNTs dispersion. Additionally, s-SWNT film showed no detectable band in the MIR region (< 0.1 eV) while the **SDOC**-dispersed film showed obvious absorption there. In literatures [25,26], most SWNTs possess one-dimensional plasmon resonance in the MIR region even after the sorting of s-SWNTs. Considering that the plasmon resonance is generated by the collective motion of free carriers [27], we suppose that the doping level of s-SWNT films obtained here is low in comparison with literatures.

### 3.2. Doping effects on electronic structures

The quantitative evaluation of both the interband transition and plasmon resonance is useful for primarily pursuing the progress of doping (Figure 2(a)). Considering its extraordinary electrochemical stability [13,28,29], AgTFSI, a typical one-electron oxidizer (hole dopant) [30], was used for a p-type dopant. The addition of AgTFSI gradually changed the spectra, achieving (1) the complete suppression of the S_11_ band (0.6–0.9 eV), and (2) the significant evolution of one-dimensional plasmon resonance in the MIR region (< 0.6 eV). These changes were dependent on the amount of AgTFSI, which enables a gradual and controlled increase in hole carrier density. Additionally, the efficiency of doping was found to depend on the thickness; thinner films preferentially underwent the doping (Figure 2(b)). The diffusion of AgTFSI seems to limit the doping process in thicker SWNT films. Control experiments showed that the amount of polymer residues, evaluated by the UV-vis absorption of **PF12**, is not a dominant factor for doping (Figure S2).

### 3.3. Thermoelectric transport of PF12/s-SWNT films

Then, the thermoelectric transport in the films of polyfluorene-dispersed SWNTs were systematically examined. Thermoelectric transport was examined using electrical conductivity (*σ*) and the Seebeck coefficient (α). Using carrier density (*n*), an elemental charge (*e*), mobility (*μ*), observed voltage (Δ*V*) and temperature difference (Δ*T*), these parameters are described as

(1) σ=neμ

(2) $$\alpha =-\frac{\Delta V}{\Delta T}$$

Furthermore, a power factor (PF), power generation ability, is described as

(3) $$PF=\alpha^{2}\sigma$$

First, an as-produced **PF12/s-SWNT-160** film showed a low electrical conductivity of ~ 0.15 S cm^−1^ (Figure 3(a)). Slight doping with 0.1 mg mL^−1^ AgTFSI dramatically increased electrical conductivity to 7.8 S cm^−1^, and the reduction of film resistivity down to less than a mega-ohm enabled the reliable measurement of the Seebeck coefficient. The order of the obtained Seebeck coefficient (~ 235 μV K^−1^) corresponds approximately to the theoretical value of slightly doped s-SWNTs [19]. Using identical feedstock films, we examined the doping effect on the thermoelectric transport. After doping (0.1–8.0 mg mL^−1^), the film showed a gradual increase in the electrical conductivity up to 1475 S cm^−1^, and a corresponding decrease in the Seebeck coefficient from a few hundreds to 41 μV K^−1^. As a result, the optimal power factor of **PF12/s-SWNT-160** was estimated at 412 μW m^−1^ K^−2^. These values are reasonable compared to those of the state-of-the-art literature [22].

The same procedure was adopted to the other four samples (**PF12-300/s-SWNT, PF12/s-SWNT-340, PF12/s-SWNT-380**, and **PF12/s-SWNT-540**). Considering the absorption spectra (Figure 2(b)), the doping proceeded preferentially for the thinner films. Accordingly, maximized electrical conductivity increased when film thickness decreased. For example, in the same doping condition, the **PF12/s-SWNT-160** film showed the conductivity of 1475 S cm^−1^, while **PF12/s-SWNT-300** and **PF12/s-SWNT-540** films showed 1151 S cm^−1^ and 857 S cm^−1^, respectively (Figure 3(b,c)). Correspondingly, a decrease in the film thickness from 380 nm down to 160 nm resulted in an increase in the power factor from 250 to 412 μW m^−1^ K^−2^. It should be noted that the power factor is mostly similar between 300 and 540 nm (Figure 3(d)). Further decrease in film thickness was technically difficult.

### 3.4. Properties of P3DT/s-SWNTs and dependence on film thickness

We further studied the thermoelectric properties of **P3DT/s-SWNT** films for comparison with **PF12/s-SWNT** films. Polythiophenes are also known as an extractant for s-SWNTs [31]. The volume content of **P3DT** in the **P3DT/s-SWNT** films was roughly evaluated using the absorption band in the visible range (extinction coefficient~6 × 10^4^ cm^−1^) at about 28 ± 6% [32], which is similar to the polymer volume fraction in **PF12/s-SWNT** films. NIR absorption spectra indicate that the diameter and doping level of s-SWNTs were likely to be similar between **P3DT/s-SWNT** and **PF12/s-SWNT** films (see Figure S3). In other words, two conducting polymers could well operate for extracting the completely un-doped SWNTs in the same diameter range and chirality distribution.

The **P3DT/s-SWNT** films of different thickness showed similar conductivity and Seebeck coefficient in the non-doped state, ca. 30–60 S cm^−1^ and ca. 150 μV/K, respectively (Figure 4(a)). Upon AgTFSI treatment, conductivity increased and the Seebeck coefficient decreased. Interestingly, we could observe no significant thickness dependence in these properties and additionally, in the optimized PF (Figure 4(b)). This thickness independence is in contrast to the results of **PF12/s-SWNT** films, despite the same alkyl chains, the similar extraction selectivity of s-SWNTs and the similar amounts of remaining polymers in both films. We then examined the oxidative doping of both conducting polymers with AgTFSI in butanol. **P3DT** exhibited the clear progression of the absorption band corresponding to the polarons state upon the treatment of AgTFSI. In contrast, **PF12** showed no significant spectral change (see Figure S4). Considering these spectrum features, the oxidation of s-SWNTs in the **PF12/s-SWNT** films is likely to occur via direct electron transfer between Ag^+^ ion and s-SWNTs. On the other hand, the doping can be mediated by the redox reaction of surrounding **P3DT** in **P3DT/s-SWNT** films. With this clear difference in the reactivity of AgTFSI in both films, the thickness effects in **PF12/s-SWNT** films are attributed to the low permeation of AgTFSI through the highly hydrophobic **PF12** layer, especially when the layer is relatively thick, and to the suppressed diffusion of AgTFSI within the layer. In contrast, **P3DT** is easily oxidized and becomes hydrophilic after reaction with AgTFSI, which promotes dopant diffusion into the deep inside of films.

Considering the conductivity and Seebeck coefficients of doped films, the maximum doping level of s-SWNTs in **PF12/s-SWNT** films should be higher than the doping level in **P3DT/s-SWNT** films. Although the reason for this difference is yet unclear, we attribute it to the role of oxidized **P3DT** and neutral **PF12** in both films. It is possible that the doping treatment with AgTFSI keeps the s-SWNTs surrounded closely by the positively charged **P3DT** chains. The closely contacting and insufficiently compensated positive charges on **P3DT** could suppress the excess doping of s-SWNT. Conversely, the ‘insulating’ [33] **PF12** layer wrapping s-SWNTs may weaken the repulsive interactions between positively charged s-SWNTs. Consequently, this buffering could support the higher maximum doping level of thin **PF12/s-SWNT** films.

## 4. Conclusion

We have revealed the thickness-dependent thermoelectric transport in **PF12**-functionalized s-SWNT films with the power factor up to 412 μW m^−1^ K^−2^, which could contribute to future thermoelectric generator design [34]. This work provides insight into chemical doping and transport in one-dimensional semiconductor networks. Particularly, film thickness should be considered in the analysis of transport in insulator-wrapped s-SWNT films. Our results could be used in materials design strategies including surfactant removal [35,36], dopant miscibility [37], and thin film integration [38].
