# Supplementary material for: Thickness-dependent thermoelectric power factor of polymer-functionalized semiconducting carbon nanotube thin films
Source: Sci Technol Adv Mater. 2018 Aug 13;19(1):581–7. doi: 10.1080/14686996.2018.1500851 (PMC6095011; doi:10.1080/14686996.2018.1500851)
Supplement: Supplemental Material [file TSTA_A_1500851_SM9592.pdf]

Supplemental materials for Nonoguchi et al “Thickness-dependent thermoelectric power factor of polymer-functionalized semiconducting carbon nanotube thin films”

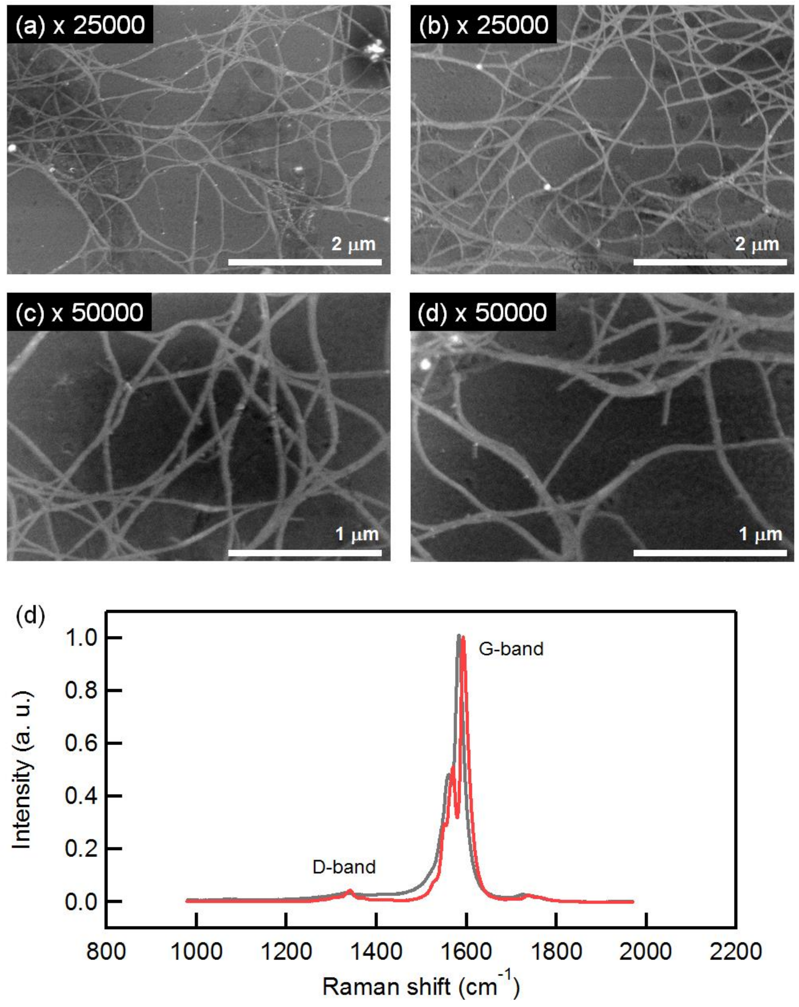

Figure S1. SEM images of PF12-functionalized s-SWNTs deposited on Si/SiO(90nm) substrates at (a, b) 25,000 and (c, d) 50,000 magnification. An applied voltage was set at 10 kV. Due to the charge-up of SWNTs, their diameters looked much larger. (e) Raman spectra of as-received (grey) and PF12-functionalized (red) s-SWNT films.

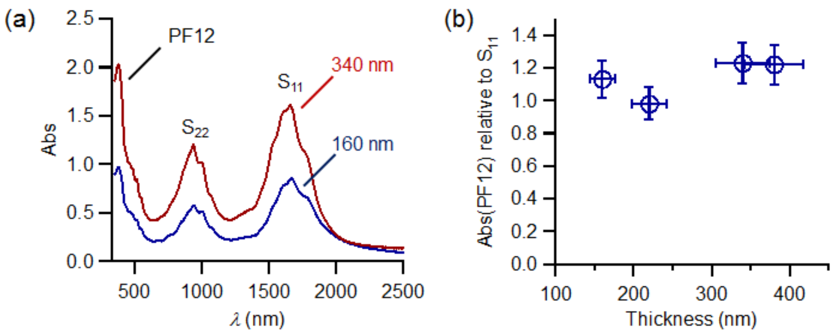

Figure S2 (a) Absorption spectra of PF12-functionalized s-SWNT thin films of different diameters. (b) Peak top ratios between the first transitions of PF12 and s-SWNTs (S_{11}).

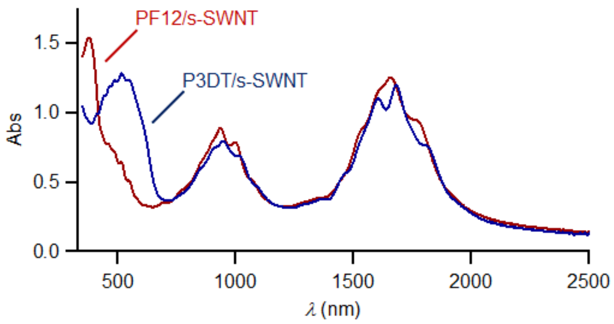

Figure S3 (a) Absorption spectra of **PF12/s-SWNT** and **P3DT/s-SWNT** thin films.

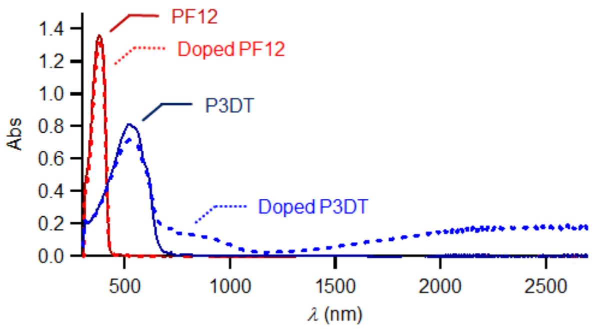

Figure S4 Absorption spectra of **PF12** (red) and **P3DT** (blue) thin films before and after the doping with 2.5 mg ml^{-1} AgTFSI butanol solution.

Table S1. SWNT films doped with the 3.0 mg mL^{-1} butanol solution of AgTFSI.

Condition	σ (S cm^{-1})	α (μV K^{-1})
As-prepared	**480**	**79**
Nine-month	**86**	**122**
